# Biallelic truncating variants in children with titinopathy represent a recognizable condition with distinctive muscular and cardiac characteristics: a report on five patients

**DOI:** 10.3389/fcvm.2023.1210378

**Published:** 2023-07-27

**Authors:** Anwar Baban, Marianna Cicenia, Monia Magliozzi, Giovanni Parlapiano, Marco Cirillo, Giulia Pascolini, Fabiana Fattori, Maria Gnazzo, Pasqualina Bruno, Lorenzo De Luca, Luca Di Chiara, Paola Francalanci, Bjarne Udd, Aurelio Secinaro, Antonio Amodeo, Enrico Silvio Bertini, Marco Savarese, Fabrizio Drago, Antonio Novelli

**Affiliations:** ^1^ Pediatric Cardiology and Arrhythmia/Syncope Complex Unit, Bambino Gesù Children’s Hospital, IRCCS, Rome, Italy; ^2^ Laboratory of Medical Genetics, Translational Cytogenomics Research Unit, Bambino Gesù Children’s Hospital, IRCCS, Rome, Italy; ^3^ Department of Imaging, Bambino Gesù Children’s Hospital, IRCCS, Rome, Italy; ^4^ Unit of Muscular and Neurodegenerative Disorders, Bambino Gesù Children’s Hospital, IRCCS, Rome, Italy; ^5^ Translational Cytogenomics Research Unit, Bambino Gesù Children’s Hospital, Rome, Italy; ^6^ Department of Cardiac Surgery, Cardiology, Heart and Lung Transplantation, Bambino Gesù Children’s Hospital, IRCCS, Rome, Italy; ^7^ Pediatric Cardiac Intensive Care Unit, Department of Cardiology and Cardiac Surgery, Bambino Gesù Children’s Hospital, IRCCS, Rome, Italy; ^8^ Department of Pathology, Bambino Gesù Children’s Hospital and Research Institute, IRCCS, Rome, Italy; ^9^ Folkhälsan Research Center, Helsinki, Finland; ^10^ Department of Medical Genetics, Medicum, University of Helsinki, Helsinki, Finland; ^11^ Department of Neurology, Vaasa Central Hospital, Vaasa, Finland; ^12^ Advanced Cardiothoracic Imaging Unit, Bambino Gesù Children’s Hospital, IRCCS, Rome, Italy; ^13^ Heart Failure and Transplant, Mechanical Circulatory Support Complex Unit, Bambino Gesù Children’s Hospital, IRCCS, Rome, Italy

**Keywords:** titinopathy, titin (*TTN*), truncating variant, dilated cardiomyopathy, neuromuscular disease, heart failure, children

## Abstract

**Background:**

Monoallelic and biallelic *TTN* truncating variants (*TTNtv*) may be responsible for a wide spectrum of musculoskeletal and cardiac disorders with different age at onset. Although the prevalence of heterozygous *TTNtv* is relatively high in the general population, cardiac phenotyping (mainly cardiomyopathies, CMPs) in biallelic titinopathy has rarely been described in children.

**Methods:**

We reviewed the medical records of pediatric patients with biallelic *TTNtv* and cardiac involvement. Clinical exome sequencing excluded pathogenic/likely pathogenic variants in major CMP genes.

**Results:**

Five pediatric patients (four male) with biallelic *TTNtv* were included. Major arthrogryposis multiplex was observed in four patients; no patient showed intellectual disability. At a cardiac level, congenital heart defects (atrial and ventricular septal defects, *n* = 3) and left ventricular non-compaction (*n* = 1) were reported. All patients had dilated cardiomyopathy (DCM) diagnosed at birth in one patient and at the age of 10, 13, 14, and 17 years in the other four patients. Heart rhythm monitoring showed tachyarrhythmias (premature ventricular contractions, *n* = 2; non-sustained ventricular tachycardia, *n* = 2) and nocturnal first-degree atrio-ventricular block (*n* = 2). Cardiac magnetic resonance (CMR) imaging was performed in all patients and revealed a peculiar late gadolinium enhancement distribution in three patients. HyperCKemia was present in two patients and end-stage heart failure in four. End-organ damage requiring heart transplantation (HT) was indicated in two patients, who were operated on successfully.

**Conclusion:**

Biallelic *TTNtv* should be considered when evaluating children with severe and early-onset DCM, particularly if skeletal and muscular abnormalities are present, e.g., arthrogryposis multiplex and congenital progressive myopathy. End-stage heart failure is common and may require HT.

## Introduction

The *TTN* gene (OMIM 188840) is located on chromosome 2q31 and consists of 363 coding exons. Titin is the largest protein in nature and a major component of muscle sarcomere. Its structure is composed of 27,000–33,000 amino acids in length with an estimated molecular weight ranging from 2,900 to 3,800 kDa (1, 2). The molecular structure of titin is divided into four regions: the N-terminal Z-disc (exon 2 to 15), the I-band (exon 29 to 152), and A-band regions (exon 153 to 358) and the C-terminal M-line, encoded by the last six exons of the *TTN* gene (358–363 or Mex1 to Mex6) (3–5).

*TTN* variants are responsible for congenital and/or progressive skeletal and/or myocardial muscle disorders. However, determining pathogenic variants remains challenging due to gene size and complex repetitive gene structure. In addition, the number of individuals who carry a rare non-pathogenic *TTN* variant is relatively high. A wide spectrum of these conditions exists, depending on the mode of inheritance (mono- or biallelic variants), age of onset (congenital, juvenile, adult onset), severity, progression, and pattern of muscles involved. Several studies have reported a potential genotype–phenotype correlation, including variants involving exon 364 and 344 leading to adult-onset autosomal dominant tibial muscular dystrophy (TMD, 600334) and adult-onset hereditary myopathy with early respiratory failure (HMERF, 603689), respectively. All remaining *TTN*-related skeletal myopathies are biallelically inherited (recessive in nature) (6).

In the field of dilated cardiomyopathies (DCMs), a rapidly increased knowledge on variants in approximately 100 genes as the cause of pathological cardiac remodeling is known. The mutation of proteins involved in many subcellular systems and different pathways leads to development of this disorder. However, phenotypic expression is variable and depends on several factors such as the type of mutated protein, the coexistence of other genetic variants, age, and life style as well (7). In particular, biallelic or digenic variants are responsible for more severe phenotypes and earlier onset of the diseases as reported in literature (8–10).

Patients carrying biallelic *TTN* truncating variants (*TTNtv*) within N2B and N2BA encoding exons seem to have an increased risk for both congenital heart defects and early cardiac involvement with development of dilated cardiomyopathy (DCM) or arrhythmic events (6).

Monoallelic *TTNtv* is not uncommon in the general population, and many data in the literature describe its association with myopathy and/or cardiomyopathy (CMP) in young individuals and adults. Moreover, Cannatà et al. reported that late-onset DCMs are more frequently associated with *TTNtv.* This context might be the example of interaction between genotype and environment as it can be influenced by hormonal, molecular, and metabolic mechanisms, lifestyle, and aging as well (11–13).

Biallelic *TTNtv* in childhood onset myopathy is extensively described, whereas deep cardiac phenotyping of individuals with biallelic *TTNtv* is less well established. While there are several reports regarding monoallelic *TTNtv* cardiac phenotyping, including arrhythmic and imaging deep phenotyping, only very few studies have described these findings in cohorts of biallelic *TTNtv*. In this study, we included a comprehensive multidisciplinary description of a cohort of 5 patients carrying biallelic *TTNtv*.

The present study includes cardiac diagnostics starting from heart rhythm, CMR imaging, and, in two individuals, histopathological specimens from explanted hearts. To the best of our knowledge, this is the first detailed description of cardiac involvement in biallelic *TTNtv* in children with titinopathy.

## Methods

This is a single-center, observational, retrospective study carried out at a tertiary academic pediatric hospital (Bambino Gesù Children's Hospital, IRCCS). We reviewed the medical records of pediatric patients with *TTN* variants in compound heterozygous (biallelic) variants and cardiac involvement seen at our Pediatric Cardiac Surgery, Cardiology, Heart and Lung Transplant Department. The study protocol conforms to ethical guidelines of the 1975 Declaration of Helsinki, as reflected in *a priori* approval by the Institution's Human Research Committee. Informed consent was obtained from the probands or their parents or legally authorized representatives also for images to be published. Data collection included demographics; family history; prenatal, perinatal, and developmental milestones; neurological, neuromuscular, and muscular magnetic resonance imaging (MRI), biopsy; and electromyography (whenever available). Cardiac data included New York Heart Association (NYHA) class, electrocardiogram (ECG), echocardiography, cardiac MRI, Holter ECG monitoring, and cardiac histopathology findings (the latter in two transplanted patients). The patient outcome measure was the incidence of major adverse cardiac events defined as death, cardiac arrest, or heart transplantation (HT). Cardiac assessment including physical examination, ECG, and echocardiography was performed in all parents of affected individuals and in their siblings whenever possible.

### Genetic analysis

Genetic testing was performed at the Genetic Laboratories of the Bambino Gesù Children's Hospital, IRCCS. DNA was extracted from peripheral blood with Qiagen columns (QIAamp DNA Minikit; Qiagen, Hilden, Germany) according to the manufacturer's instructions. Concentration and purity of DNA samples were quantified by ND-1000 spectrophotometer (NanoDrop; Thermo Scientific, Waltham, MA, USA) and by FLx800 Fluorescence Reader (BioTek, Winooski, VT, USA).

#### Next-generation sequencing analysis and variant interpretation

Next-generation sequencing (NGS) analysis was performed on genomic DNA using the Twist Custom Panels (Clinical Exome Twist Bioscience) according to the manufacture's protocol on Illumina NexSeq550 or NovaSeq6000 platform (Illumina, San Diego, CA, USA). The reads were aligned to human genome build GRCh37/UCSC hg19. The Dragen Enrichment application of BaseSpace (Illumina) and the TGex software (LifeMap Sciences, Alameda, CA, USA)—Geneyx Analysis (knowledge-driven NGS analysis tool powered by the GeneCards Suite) were used for variant calling and annotating variants, respectively. Gene variants associated with CMP gene were scored and filtered by the TGex-Geneyx analysis software. Among the evaluated variants by TGex-Geneyx and matching with the CMP phenotype, those meeting the following parameters were filtered: (1) non-synonymous exonic or ±5 bp intronic variants, (2) minor allele frequency in the Genome Aggregation Database (GnomAD) of less than 0.01 (1%), (3) quality of the call variant: coverage of ≥30X and GQ of ≥50, and (4) at least 20% of reads showing the alternative allele (Alt of >20%). Variants were visualized by the Integrative Genomics Viewer. Sequence data were carefully analyzed, and the presence of all suspected variants was checked in the public databases (gnomAD, dbSNP, 1,000 Genomes Project, EVS, ExAC). An *in silico* prediction of variants’ pathogenicity was obtained using Sorting Intolerant From Tolerant (SIFT), Polymorphism Phenotyping v2 (PolyPhen-2), and MutationTaster for the prediction of deleterious non-synonymous single nucleotide variant for human diseases. The variants were evaluated by VarSome (14) and classified according to the American College of Medical Genetics and Genomics (ACMG) criteria (15).

*TTN* variants are annotated on the inferred-complete meta-transcript (NM_001267550.1). The variant nomenclature has been verified using Mutalyzer 3. Cardiac proportion spliced in (PSI) was annotated as previously reported on cadiodb.org.

The singleton's variants were confirmed by Sanger sequencing following a standard protocol (BigDye Terminator v3.1 Cycle Sequencing Kit, Applied Biosystems by Life Technologies). Segregation analysis was performed in all parents of each patient. Moreover, none of the analyzed unaffected siblings showed biallelic inheritance of the variants.

#### CMP gene list

The following is a CMP gene list: *ABCC9*, *ANK2*, *ALPK3*, *ANKRD1*, *ACTC1*, *ACTN2*, *BAG3*, *CACNA1C*, *CALM1*, *CALM2*, *CALM3*, *CALR3*, *CAV3*, *CTNNA3*, *CSRP3*, *DES*, *DNAJC19*, *DOLK*, *DSC2*, *DSG2*, *DSP*, *DTNA*, *EMD*, *EYA4*, *FAH*, *FHL2*, *FHL1*, *FKRP*, *FKTN*, *FLNC*, *FOXRED1*, *FXN*, *GATA4*, *GATA5*, *GATA6*, *GATAD1*, *GFM1*, *GLA*, *GLB1*, *GNPTAB*, *GUSB*, *GYG1*, *HCN4*, *HFE*, *HRAS*, *ILK*, *JARID2*, *JPH2*, *JUP*, *KCNE1*, *KCNE2*, *KCNH2*, *KCNJ2*, *KCNJ5*, *KCNJ8*, *KCNQ1*, *KLHL24*, *KRAS*, *LAMA2*, *LAMA4*, *LAMP2*, *LDB3*, *LIAS*, *LMNA*, *LZTR1*, *MAP2K1*, *MAP2K2*, *MLYCD*, *MRPL3*, *MRPL44*, *MRPS22*, *MTO1*, *MYBPC3*, *MYOM1*, *MYOT*, *MYOZ2*, *MYPN*, *MYH6*, *MYH7*, *MYL2*, *MYL3*, *NEBL*, *NEXN*, *NF1*, *NONO*, *NRAS*, *OBSCN*, *PDLIM3*, *PKP2*, *PLD1*, *PLN*, *PMM2*, *PPA2*, *PPCS*, *PRDM16*, *PRKAG2*, *PTPN11*, *QRSL1*, *RAF1*, *RBM20*, *RIT1*, *SCN4B*, *SCN5A*, *SCO2*, *SDHA*, *SGCD*, *SGCG*, *SHOC2*, *SLC22A5*, *SLC25A3*, *SNTA1*, *SOS1*, *SPEG*, *SURF1*, *TAFAZZIN*, *TBX20*, *TCAP*, *TGFB3*, *TMEM43*, *TMEM70*, *TNNC1*, *TNNI3*, *TNNT2*, *TPM1*, *TRDN*, *TRIM63*, *TTN*, *TTR*, and *VCL*.

### Histology and immunohistochemistry

#### Cardiac specimens

Myocardial samples were obtained either from right ventricular (RV) endomyocardial biopsy or left ventricular (LV) myocardium at the time of LV assist device placement or HT. Myocardial samples were processed according to standard histology protocols for hematoxylin and eosin and Masson's trichrome.

#### Cardiac magnetic resonance imaging

Comprehensive biventricular assessment with CMR requires multiple imaging planes, starting with conventional 2D long-axis cine-balanced steady-state free precession images, particularly including RV two-chamber view and short-axis cine views from base to apex.

We also performed segmented 2D high-resolution late gadolinium enhancement (LGE) in LV/RV long-axis view (including outflow tract views) and short-axis stack.

#### Muscular magnetic resonance imaging

MRI coronal and axial T1-weighted and T2 sequences were obtained; isotropic 3D T1 vibe DIXON images (millimetric voxel) were obtained in patients 1 and 4.

## Results

We report on five pediatric patients (four males) with molecularly confirmed biallelic *TTNtv* in trans with a classification according to ACMG class 4 and class 5 (likely pathogenic and pathogenic variants) (16).

At the cardiac level, congenital heart defects (atrial and ventricular septal defects) were seen in three patients requiring closure in two; LV non-compaction (LVNC) was detected in one patient. All patients had DCM diagnosed at birth in one patient and at the age of 10, 13, 14, and 17 years in the other patients. Heart rhythm monitoring showed tachyarrhythmias [premature ventricular contractions (PVCs), *n* = 2; non-sustained ventricular tachycardia (NSVT), *n* = 2); and nocturnal first-degree atrio-ventricular block (AVB), *n* = 2]. CMR was performed in all patients, showing peculiar LGE distribution in three patients. As expected, hyperCKemia is not constant in titinopathies. Four patients developed end-stage heart failure. End-organ damage occurred in two patients who underwent successful HT.

**Figure 1:**
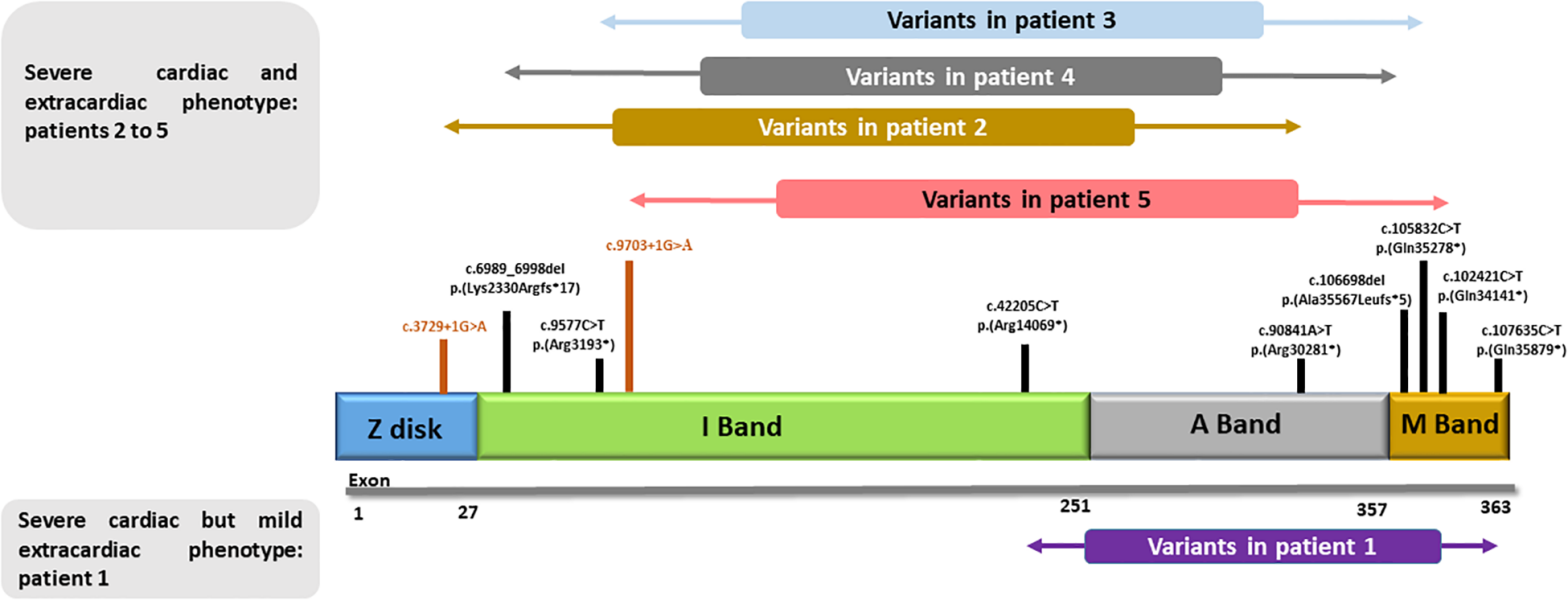
Schematic structure of titin: principal cardiac long isoform N2BA (NM_001256850.1) and schematic representation of the variants presented in our cohort. Patient 1, with very mild musculoskeletal phenotype, carries variants that are located beyond the second half of the I-band and the most distal part of the M-band. All other patients, with remarkable extracardiac involvement, show more proximally located variants.

**Figure 2:**
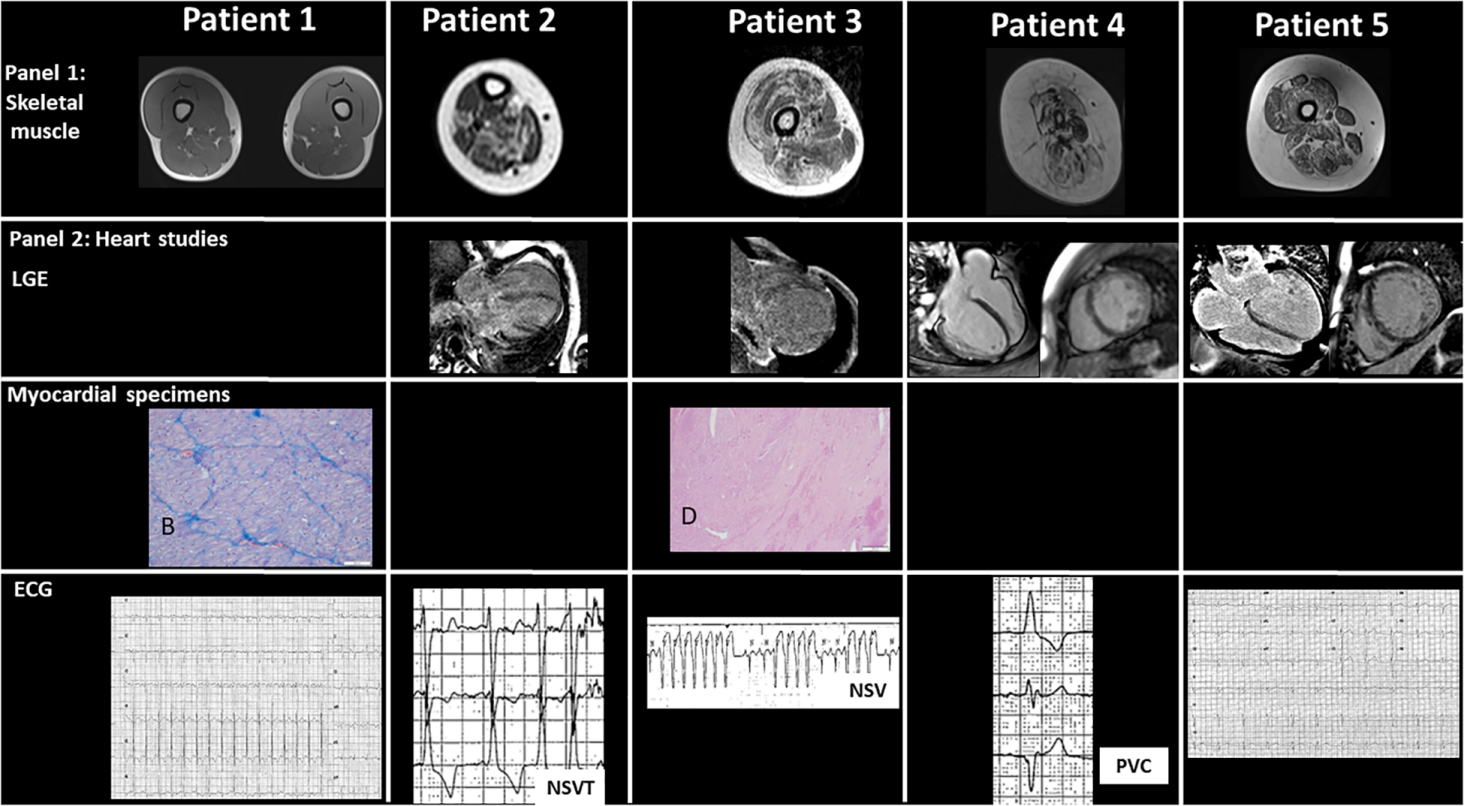
Panel 1 shows skeletal muscular magnetic resonance imaging with diffuse fibrofatty infiltration of different degree. Minimal changes are observed in patient 1, while changes in almost each muscular segment are present in patient 4. Panel 2 includes major cardiac studies including cardiac magnetic resonance imaging in patients 2–5 with variable degree of late gadolinium enhancement of different intensity in three out of five patients, mainly in the subepicardial region. Specific histological specimens from extracted hearts of patients 1 and 3 are included: (**A**) in patient 1, the myocardium is characterized by polymetric and polymorphic nuclei of cardiomyocytes (HE, 10×). (**B**) Masson trichrome staining shows mild fibrotic expansion of the interstitium (10×), while in patient 3, (**C**) eccentric hypertrophy of the left ventricular free wall can be seen (HE, 1.25×). (**D**) In the insert, at higher magnification, large areas with loss of myocardium replaced by fibrosis (HE, 40×). The lower panel shows the ECG changes, including non-sustained ventricular tachycardia and premature ventricular contractions in patients 2, 3, and 4, respectively.

**Figure 3:**
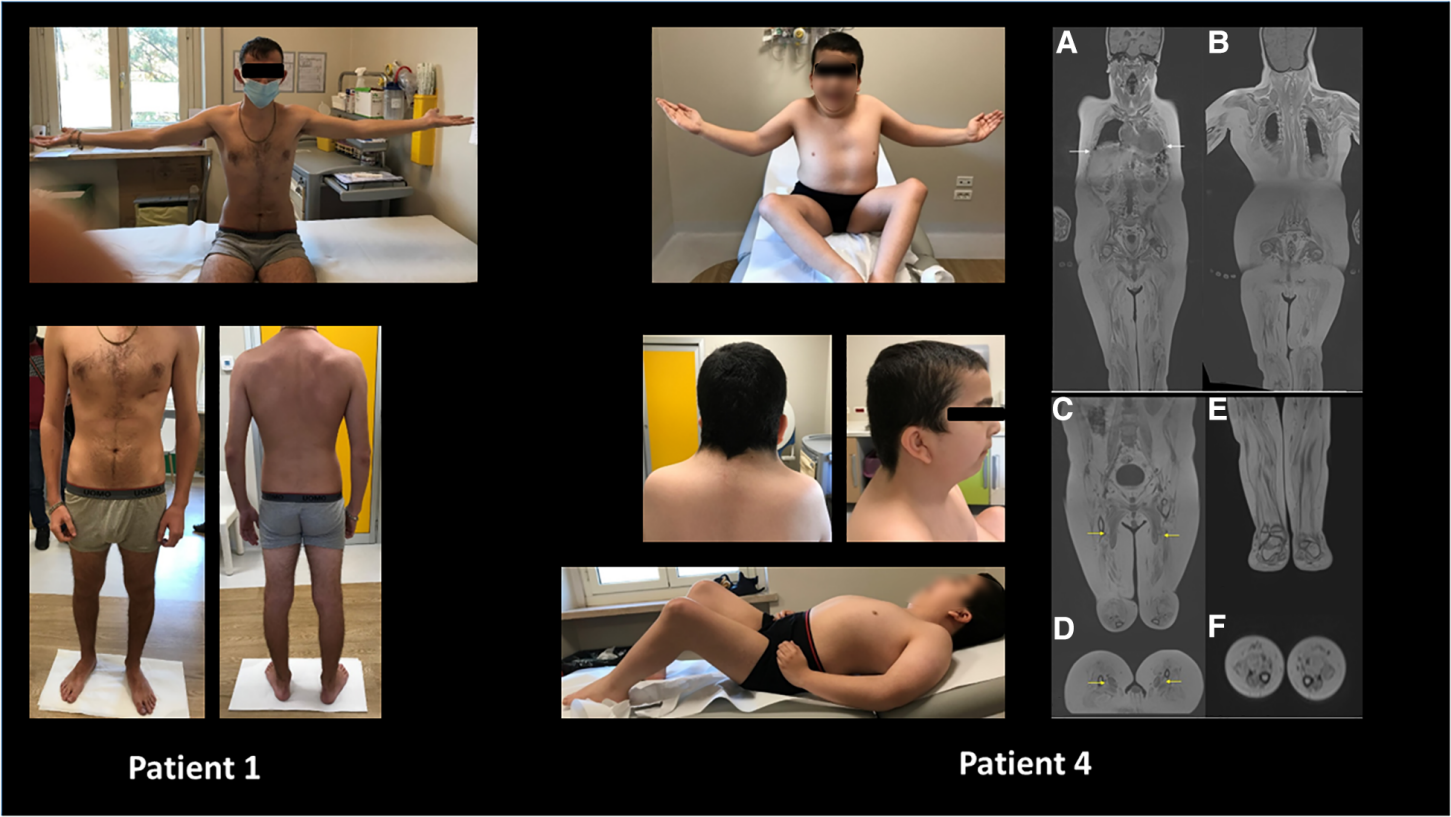
Wide phenotypic variability was noticed in the study cohort ranging from apparently no major skeletal or multisystemic involvement (patient 1) to major arthrogryposis, muscular rigidity, and craniofacial changes in patient 4. Moreover, in the latter, panoramic coronal curved multiplanar reconstructions (**A,B**) show diffuse, severe, end-stage fatty infiltration of all muscular groups, including intercostal ones (white arrows). Coronal and reformatted axial VIBE T1 DIXON of pelvis and thighs (**C,D**) revealed a less evident (moderate) bilateral fatty infiltration of adductor longus muscles (yellow arrows). Diffuse severe fatty infiltration of all muscular groups of the pelvis, thighs, and legs. Coronal and reformatted axial VIBE T1 DIXON (**E,F**) of the legs shows diffuse end-stage fatty infiltration of all muscular groups.

**Figure 4:**
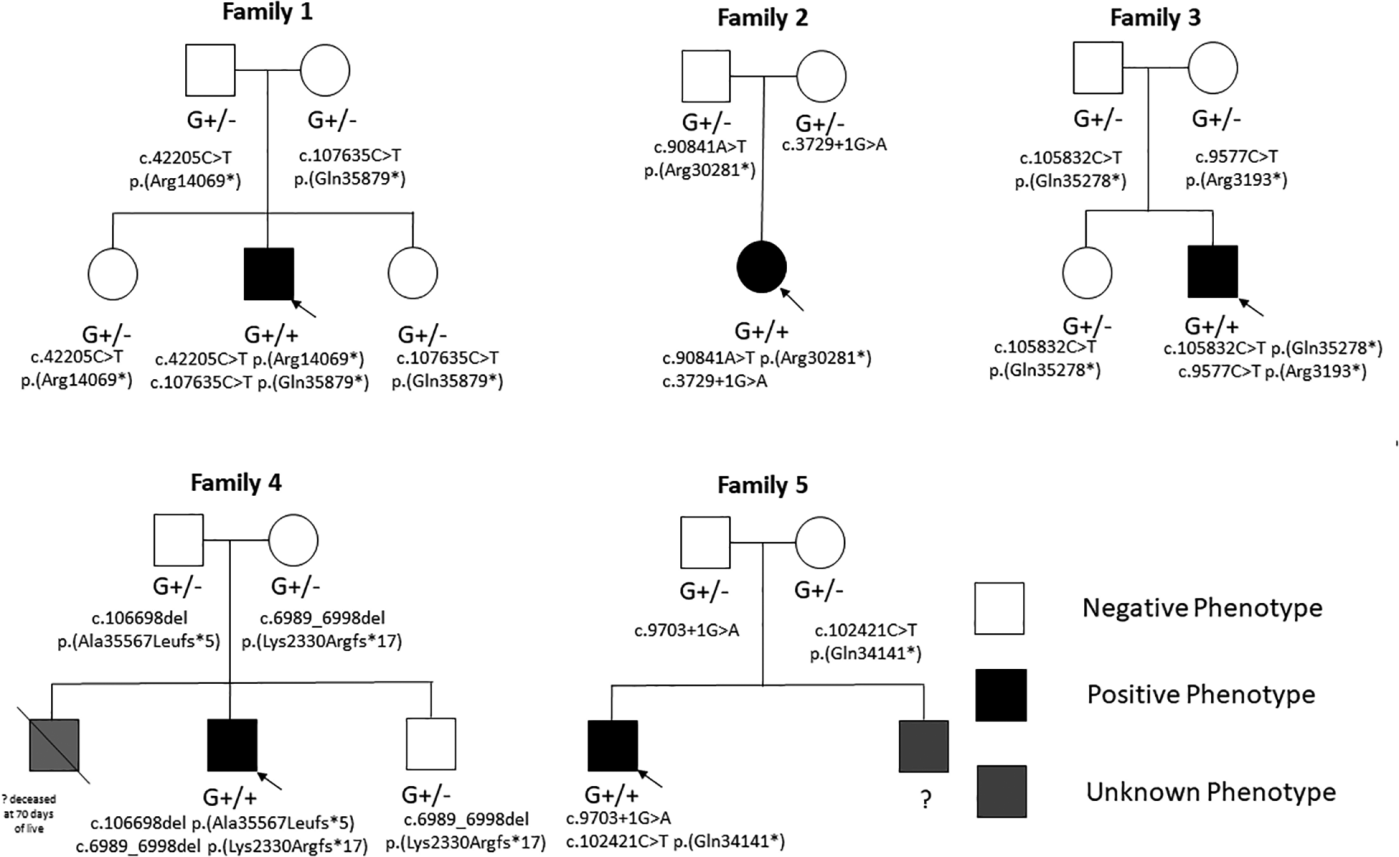
Family pedigrees of the study cohort showing phenotype-negative individuals with heterozygous variants and phenotype positive individuals with biallelic variants.

At a systemic level, major arthrogryposis multiplex was present in four patients, whereas no patient showed intellectual disability (Table 1, Figures 1–4).

**Table 1:** Description of the study cohort: phenotypic and genotypic characteristics, cardiac and muscular investigations, laboratory tests, and family history.

	Patient 1	Patient 2	Patient 3	Patient 4	Patient 5
Age at first evaluation at our center	14 years for cardiac reason	At birth for cardiac + neuromuscular reasons	10 years for cardiac reason	4 years for neuromuscular reason/13 years for cardiac reason	7 years for neuromuscular reason/17 years for cardiac reason
Age at last FU (years)	19	6	21	14	18
Sex	M	F	M	M	M
Ethnical origin	Caucasian	Caucasian	Caucasian	Arab	Caucasian
Variant 1 (exon, cardiac PSI)	c.107635C > T p.(Gln35879*) (exon363, PSI = 100)	c.90841A > T p.(Arg30281*) (exon 336, PSI = 100)	c.105832C > T p.(Gln35278*) (exon 359, PSI = 100)	c.6989_6998del p.(Lys2330Argfs*17) (exon 30, PSI = 100)	c.102421C > T p.(Gln34141*) (exon 359, PSI = 100)
Variant 2 (exon, cardiac PSI)	c.42205C > T p.(Arg14069*) (exon 231, PSI = 100)	c.3729 + 1G > A (exon 22, PSI = 100)	c.9577C > T p.(Arg3193*) (exon 41, PSI = 100)	c.106698del p.(Ala35567Leufs*5) (exon 359, PSI = 100)	c.9703 + 1G > A (exon 41, PSI = 100)
Type of variant	Variant 1: non-sense Variant 2: non-sense	Variant 1: non-sense Variant 2: splice site	Variant 1: non-sense Variant 2: non-sense	Variant 1: frameshift Variant 2: frameshift	Variant 1: non-sense Variant 2: splice site
ACMG classification	Variant 1: LP (PVS1, PM2) Variant 2: LP (PVS1, PM2)	Variant 1: LP (PVS1, PM2) Variant 2: LP (PVS1, PM2)	Variant 1: LP (PVS1, PM2) Variant 2: LP (PVS1, PM2)	Variant 1: LP (PVS1, PM2) Variant 2: LP (PVS1, PM2)	Variant 1: LP (PVS1, PM2) Variant 2: LP (PVS1, PM2)
Frequency (gnomAD exome)	Variant 1: 0.0000121 Variant 2: not found	Variant 1: not found Variant 2: not found	Variant 1: not found Variant 2: not found	Variant 1: not found Variant 2: not found	Variant 1: not found Variant 2: not found
Inheritance	Variant 1: maternal Variant 2: paternal	Variant 1: paternal Variant 2: maternal	Variant 1: paternal Variant 2: maternal	Variant 1: maternal Variant 2: paternal	Variant 1: maternal Variant 2: paternal
ECG	SR	SR, VR abnormalities	SR	SR, negative T-waves in infero-lateral leads	SR, VR abnormalities, low-voltage limb leads
24 h Holter ECG	Frequent polymorphic PVCs (burden 1%), sporadic SVPCs	NSVT (max 4 beats) Nocturnal first-degree AVB; QTc at upper limit of normal	NSVT	Rare dimorphic PVCs, QTc at upper limit of normal	No arrhythmias Nocturnal first-degree AVB
Echocardiography	LVEF 11% and severe LV dilatation (EDD 69 mm, Z score 6.8) Severe RV dysfunction Moderate MR and TR	LVEF 15%–20% LV diameters at upper limit of normal LVEF at last FU 45%	LVEF 15% Severe LV dilatation Diastolic dysfunction LVNC	Mild LV dilatation (EDD 49 mm, Z score 2.2), LVEF 36% Hypertrabeculation	Biventricular dilatation (EDD LV 66 mm, Z score 4.1), LVEF 25%–30% Moderate MR
CMR	No LGE	Subtle LGE changes	LGE: transmural IVS	Subepicardial linear LGE along LV lateral wall	Transmural LGE, LV lateral wall thinning/T1 mapping: interstitial fibrosis
Highest BNP level (pg/ml)	2,790.1	359	863	1,132	6,500
Highest hs-TnI level (pg/ml)	122.1	4.9		17.4	99
Highest CPK level (U/l)	Normal	Normal	1,396 post BH	Normal	4,713
Histological heart specimens	No signs of acute myocarditis Myocardial cells with polymetric and polymorphic nuclei and hydropic cytoplasmic degeneration and interstitial fibrosis	Not performed	Polymetric, polymorphic cells, cytoplasmic vacuolization, disarray, and diffuse fibrosis	Not performed	Not performed
Cardiac symptoms	Yes (acute heart failure)	Yes (acute heart failure), Growth delay	Yes (acute heart failure)	Mild dyspnea	Y (acute heart failure)
NYHA class/Ross	IV	II–III	III–IV	I–II	II
Congenital heart defects	Small ASD	ASD and VSD surgically corrected	No	No	ASD percutaneous closure
Dilated cardiomyopathy	Yes	Yes	Yes	Yes, 13 years	Yes, 14 years
Pregnancy/birth history					
Reduced fetal movement	Unavailable	No, normal movement	Unavailable	No	Unavailable
Abnormal amniotic fluid	No	Oligoidramnios	Unavailable	Oligoidramnios	Unavailable
Additional *in utero* ultrasound anomalies	No	Increased nuchal translucency; US: perinatal reduced LVEF	Unavailable	No	Unavailable
Gestational age	At term	37 + 5	At term	At term	At term
Birth weight (kg)	2.8	2.6	2.9	3	2.7
Birth	NVD	Induced VD	CS, for previous CS	CS	CS
Abnormal presentation at delivery	No	No	No	No	No
Congenital features					
C. hypotonia/weakness	No	Generalized hypotonia	Generalized hypotonia	Hypotonia	Hypotonia
C. limb contractures	No	Bilateral fixed-flexion elbows, knees, ankles	Bilateral fixed-flexion elbows, knees, ankles	Pterygium/arthrogryposis elbow and knees	Retraction of the Achilles tendon Rigid spine
C. fractures	No	No	No	No	No
Neonatal respiratory difficulties	No	No	No	No	Yes
Neonatal feeding difficulties	No	Unavailable	Unavailable	Yes	Yes
Other congenital features	No	Clubfoot	No	Cleft palate	No
Early motor development and ambulation					
Delay at age of sitting independently	No	Yes	Yes	Yes	Yes
Delay at age of crawling	No	Yes	Yes	Yes	Yes
Delay at age of walking independently	No	Yes	Yes (3 years)	Yes	Yes (2 years)
Able to run?	Yes	No	No	No	No
Able to jump?	Yes	No	No	No	No
Gowers’ positive	No	Cannot stand without aid	Wheelchaired	Wheelchaired	Few steps without help
Pattern of limb weakness					
Severity	Absent	Severe	Severe	Severe	Moderate
Symmetrical/asymmetrical	No	Symmetrical	Symmetrical	Symmetrical	Symmetrical
Proximal/distal involvement	Normal	Both	Both	Both	Distal > proximal
Muscle bulk					
Muscular biopsy	No	Muscular dystrophy	Multicore disease dystrophy	Primary myopathy	No
Pattern of muscle hypotrophy	Dystrophic aspect	Generalized hypotonia	Generalized hypotonia	Generalized hypotonia	Generalized hypotonia
Muscles involved	Mild	Generalized, diffused pterygium-like/semiflexed elbows and knees	Generalized, diffused pterygium-like/semiflexed elbows and knees	Generalized, diffused pterygium-like/semiflexed elbows and knees	Generalized, diffused pterygium-like/semiflexed elbows and knees
Muscular MRI	Very mild changes	Fatty infiltration of legs and paravertebral muscles	Extensive osteopenia and bilateral symmetric hypotrophy with mild-to-moderate fatty infiltration Coxa valga bilaterale	Severe fibrofatty infiltration of the muscular components of almost all muscular districts including intercostal muscles	Diffuse fatty infiltration of the muscular component of both legs
Dysmorphic features	Long face, prominent maxillary bones, partial right palpebral ptosis	Plagiocephaly, prominent glabella and eyes, eversion of lower palpebra, low-set ears, wide nasal tip, downturned angles of the mouth, tendency to bifid uvula, short labial frenulum, short neck, pterygium colli, pectus excavatum, arachnodactyly, simple dermatoglyphic. Toes overriding	Long face, prominent maxillary bones, downslanted eyes, dental overcrowding, high-arched palate, microstomia	Long face, turricephaly, downslanted palpebral fissure, mild palpebral ptosis, low-set ears, synophria, flaring of lateral part of eyebrows, high nasal bridge, long philtrum, cone-shaped overcrowding of teeth, open bite, short neck, pterygium, single palmar crease, bell-shaped thorax	Long face, turricephaly, downslanted palpebral fissure, mild palpebral ptosis, low-set ears, hypotelorism, high nasal bridge, overcrowding of teeth, high-arched palate, microstomia, hair tail in retroauricular space, synophria, short neck, pterygium colli, narrow shoulders, single palmar crease, small hands, deformed toes
Vocal cord/bulbar/pharyngeal involvement					
Chewing/swallowing difficulties	No	No	No	No	No
Requirement for NGT/parenteral gastrostomy feeding	No	NGT at first month of life	No	No	No
Voice/vocal cord abnormalities	No	No	Nasal voice	Nasal voice	Nasal voice
Neck features					
Neck flexion weakness	No	Left flexion	Semifixed flexion	Semifixed flexion	Semifixed flexion
Neck extension weakness	No	No	Unavailable	Unavailable	Unavailable
Limited range of neck movement	No	Yes	Yes	Yes	Yes
Axial features	Mild scoliosis	Scoliosis, rigid spine, neck pterygium	Severe scoliosis, hyperlordosis	Severe scoliosis, hyperlordosis	Scoliosis
Respiratory insufficiency					
Frequent severe respiratory infections	Unavailable	Yes Abnormal cough reflex Mild obstructive nocturnal hypo/apnea	Yes Moderate restrictive respiratory failure	Yes Severe Abnormal cough reflex	Spirometry not valid for reduced compliance
Foot features					
Pes cavus	No	No	Unavailable	Wheelchaired	—
Pes planus	No	No	Unavailable	Wheelchaired	Clubfoot
Other foot abnormalities	No	Clubfoot	Fixed ankle movements	Wheelchaired	Severe equinus deformity
BMD *Z score*	—	−4.3 (lumbar spine)	−3 (femoral neck)	−2.3 (lumbar spine)	—
Other features					
Height/weight percentile at last observation	Ht 10–25°/wt 10–25	Ht <3°/wt 15°	Ht <3°/wt <3°	Ht <3°/wt 3–10°	Ht 94°/wt 35°
Joint hypermobility/ligamentous laxity	No	No	No	Hands and feet joint hypermobility	No
Intellectual disability	No	Yes	No	No	Suspected mild learning difficulties/exposure to smoking and drugs
Family history					
Father cardiac screening	Normal	Normal	Normal	Normal	Normal
Mother cardiac screening	Normal	Normal	Normal	Normal	Normal
Siblings cardiac screening and genotype	Normal (two sisters: heterozygous)	—	Normal (one sister: heterozygous)	Normal (one brother: heterozygous)	? Reported a brother with similar NM signs, but never available for cardiac screening
Previous abortions/early death in family history	No	No	Two previous abortions	One brother died at 70 days	

ACMG, America College of Medical Genetics; ASD, atrial septal defect; AVB, atrio-ventricular block; BH, Berlin Heart; BNP, B-type natriuretic peptide; CPK, creatine phosphokinase; CMR, cardiac magnetic resonance; CS, cesarean section; EDD, end-diastolic diameter; F, female; FU, follow-up; hs-TnI, high-sensitivity troponin I; IVS, interventricular septum; LGE, late gadolinium enhancement; LP, likely pathogenic; LV, left ventricular; LVEF, left ventricular ejection fraction; LVNC, left ventricular non-compaction; M, male; MR, mitral regurgitation; MRI, magnetic resonance imaging; NGT, nasogastric tube; NM, neuromuscular; NSVT, non-sustained ventricular tachycardia; NVD, normal vaginal delivery; NYHA, New York Heart Association; PSI, proportion spliced in; PVC, premature ventricular contraction; RV, right ventricular; SR, sinus rhythm; SVPC, supraventricular premature contraction; TR, tricuspid regurgitation; VD, vaginal delivery; VR, ventricular repolarization; VSD, ventricular septal defect; BMD, bone mineral density; TTN variants are annotated on the inferred-complete meta-transcript (NM_001267550.1). Cardiac PSI as previously reported (https://www.cardiodb.org/titin/titin_transcripts.php).

### Case presentations

#### Patient 1

Patient 1 presented at the age of 15 years with acute heart failure symptoms and was transferred to our institution where he was diagnosed with DCM with severe biventricular dilatation and dysfunction [left ventricular ejection fraction (LVEF) of 11%]. No LGE was detected on previous CMR performed at another center.

On admission to our pediatric intensive care unit (ICU), 24-h Holter ECG monitoring showed frequent polymorphic PVCs (burden of 1%) and sporadic premature supraventricular contractions (PSVCs). Due to intractable heart failure unresponsive to conventional therapy, he was offered mechanical circulatory support and subsequently HT. Histological specimens after HT showed myocardial cells with polymetric and polymorphic nuclei and hydropic cytoplasmic degeneration and interstitial fibrosis.

Clinical geneticist evaluation at the ICU confirmed dystrophic muscular mass, long face, prominent maxillary bones, partial right palpebral ptosis, and mild scoliosis. Muscular biopsy was not performed. Muscular MRI was performed at the age of 19 years showing no muscular involvement (no fatty infiltration neither edema). The patient is in stable cardiac condition undergoing regular follow-up.

Genetic investigation confirmed biallelic *TTNtv*: (1) c.107635C > T p.(Gln35879*) (exon363, PSI = 100), maternally inherited, and (2) c.42205C > T p.(Arg14069*) (exon 231, PSI = 100), paternally inherited. No other pathogenic nor likely pathogenic variants were detected by NGS for the known genes causative for CMP.

His parents and two sisters in heterozygous state showed normal cardiac screening including cardiac evaluation, ECG, and echocardiography.

#### Patient 2

Patient 2 presented at birth for large atrial and ventricular septal defects that were surgically corrected. During the first year of life, she showed failure to thrive and heart failure symptoms. Progressive biventricular dilatation and dysfunction became evident and more severe (LVEF of 15%–20%) with partial response to pharmacological therapy. CMR confirmed echocardiography findings and subtle LGE changes. During follow-up, 24-hour Holter ECG monitoring showed nocturnal first-degree AVB and NSVT.

Hypotonia was evident since the first month of life and a muscular biopsy, which was performed at another center, concluded for muscular dystrophy. Psychomotor delay was evident. Systemic features included generalized hypotonia; left fixed-flexion position of the neck; neck pterygium; flexion contracture of bilateral elbow, knee, and ankle joints; scoliosis; rigid spine; pectus excavatum; clubfoot; and overriding toes. Facial dysmorphic features included plagiocephaly, prominent glabella, external eversion of lower eyelids, low-set ears, wide nasal tip, downturned mouth corners, tendency to bifid uvula, and short labial frenulum.

Muscular MRI showed diffuse muscular hypotrophy, fatty infiltration of the legs (mild–moderate involvement of posterior muscles—soleus, gastrocnemius, flexor halluces longus, and peroneus muscles), and mild fatty infiltration of paravertebral muscles (without signs of edema) associated with severe rotoscoliosis.

At the last follow-up at 6 years of age, partial systolic function recovery was evident (borderline-preserved LVEF of 46%), with septal hypokinesis. Family history was negative for cardiac defects or CMP. Cardiac biopsy was not performed. Genetic investigations included normal microarray CGH and NGS panel for CMP confirmed biallelic *TTNtv*: (1) c.90841A > T p.(Arg30281*) (exon 336, PSI = 100), paternally inherited, and (2) c.3729 + 1G > A (exon 22, PSI = 100), maternally inherited.

Her parents in heterozygous state showed normal cardiac screening including cardiac evaluation, ECG, and echocardiography.

#### Patient 3

The proband is the second son of healthy non-consanguineous parents. He was diagnosed at the age of 10 years with acute heart failure. On admission, echocardiography showed severe LV dilatation and LVEF of 15%, diastolic dysfunction, and LVNC. CMR confirmed these findings and showed intramyocardial intraventricular septal LGE. NSVT was also recorded during in-hospital monitoring before HT. Despite maximal treatment at the ICU and due to refractory critical conditions, he was managed with ventricular assist device and subsequently with HT (at the age of 11 years). After HT, cardiac histology specimens showed polymorphic and polymetric nuclei and vacuolar cytoplasmic degeneration, interstitial and substitutive myocardial fibrosis, and arteries and arterioles with intimal hyperplasia. All these features were consistent with the clinical diagnosis of DCM.

At the systemic level, he also had a congenital myopathy with marked hypotrophy and generalized hypotonia; diffused fixed-flexion position (pterygium-like changes) at the elbows, knees, and hips; severe kyphoscoliosis, and bilateral coxa valgus. Lengthening of the bilateral Achilles tendon was performed. He also had a motor delay and became wheelchaired. Muscular lower-limb MRI performed at the age of 14 years showed diffuse osteopenia and bilateral symmetric hypotrophy with mild-to-severe fatty infiltration of the thigh muscle (both posterior and anterior compartments) and legs (mostly posterior muscles). Muscular biopsy was also performed leading to a diagnosis of “multicore disease.”

Dysmorphic features consisted in long face, prominent glabella, prominent maxillary bones, downward slanted eyes, dental overbrowning, high-arched palate, and microstomia.

NGS panel for CMP confirmed biallelic *TTNtv*: (1) c.105832C > T p.(Gln35278*) (exon 359, PSI = 100), paternally inherited, and (2) c.9577C > T p.(Arg3193*) (exon 41, PSI = 100), maternally inherited.

His parent and older sister in heterozygous state showed normal cardiac screening including cardiac evaluation, ECG, and echocardiography.

#### Patient 4

The patient came to our attention at the age of 13 years for mild dyspnea and was diagnosed with DCM due to moderate–severe LV dysfunction (borderline-preserved LVEF of 36%), hypertrabeculation of the LV lateral wall, and mild left atrial enlargement. CMR revealed subepicardial linear LGE along the LV lateral wall. At 24-hour Holter ECG monitoring, rare dimorphic PVCs and QTc at the upper limit of normal were detected during follow-up. Pharmacological therapy was started, and LV function improved (LVEF of 48% at CMR 1 year later).

At the systemic level, the patient had normal cognitive and language development; he was followed up since 4 years for short stature and generalized muscular hypotonia. Muscular biopsy performed at the age of 5 years showed signs of suspected primary myopathy with no specific signs of a definitive form of congenital myopathy. He underwent lengthening of the bilateral Achilles tendon, knee flexors, and adductors. Currently, the proband is wheelchair dependent. In early infancy, he was operated for cleft palate. Whole-body muscular MRI showed diffuse severe fatty infiltration, including intercostal muscles; in the thighs, adductor longus muscles were less involved (Figure 3). Despite the absence of frequent respiratory infections, recent respiratory function tests showed severe restrictive abnormalities and absence of cough reflex. Non-invasive respiratory ventilation was planned to be started soon.

Dysmorphic features included turricephaly, long face, downslanting palpebral fissure, mild palpebral ptosis, low-set ears, synophria, lateral eyebrow flaring, high nasal bridge, long philtrum, cone-shaped overcrowding of teeth, open cross bite, short neck, single palmar creases, bell-shaped thorax, hands and feet joint hypermobility, and pterygium/arthrogryposis involving the elbows and knees.

Genetic investigations included normal karyotyping and microarray CGH. NGS panel for CMP confirmed biallelic *TTNtv*: (1) c.6989_6998del p.(Lys2330Argfs*17) (exon 30, PSI = 100), maternally inherited, and (2) c.106698del p.(Ala35567Leufs*5) (exon 359, PSI = 100), paternally inherited.

His parents and younger brother in heterozygous state showed normal cardiac screening including cardiac evaluation, ECG, and echocardiography. Family history included an older brother who died at 70 days of life for a cardiac problem (unavailable autopsy) and a maternal grandmother who died at his 60s for DCM.

#### Patient 5

The patient came to our attention at the age of 16 years for cardiac symptoms, and diagnostic work-up revealed LVNC, with biventricular dilatation and severe biventricular dysfunction (LVEF of 25%–30%) and moderate mitral regurgitation. During infancy, he underwent percutaneous closure of an atrial septal defect. No arrhythmias were detected at 24-h Holter ECG monitoring but only a nocturnal first-degree AVB. CMR revealed transmural LGE and thinning of the LV lateral wall/alteration of T1 mapping as for interstitial fibrosis and confirmed severe biventricular dysfunction (LVEF of 22%).

At the systemic level and during the neonatal period, he suffered from hypotonia and feeding difficulties, motor delay, diffused pterygium-like/semiflexed elbows and knees, retraction of the Achilles tendon, rigid spine, scoliosis, bilateral clubfoot, severe equinus deformity of the lower limbs, mild hyposthenia of shoulder girdle, leg muscular hypotrophy, and ability to do few steps without help. Elevation of creatine phosphokinase above 4,000 U/L was also noted. Electroneurography was normal.

Dysmorphic features included long face, turricephaly, down slanting palpebral fissure, mild palpebral ptosis, low-set ears, hypotelorism, high nasal bridge, dental crowding, high-arched palate, microstomia, hair tail in retroauricular space, synophria, short neck, pterygium colli, narrow shoulders, single palmar crease, small hands, and deformed toes.

Muscular biopsy was not performed for parents’ decision. Lower-limb muscular MRI revealed diffuse fatty infiltration of the muscular component of thighs, legs, and pelvis: moderate–severe involvement of gluteus maximus, symmetric involvement (mild–moderate) of thighs (both anterior and posterior muscles), less extensive but diffuse mild fatty infiltration of the legs, and minimal muscular edema both in thighs and legs.

Genetic investigations included normal microarray CGH and NGS panel for cardiomyopathy confirmed biallelic *TTNtv*: (1) c.102421C > T p.(Gln34141*) (exon 359, PSI = 100), maternally inherited, and (2) c.9703 + 1G > A (exon 41, PSI = 100), paternally inherited.

His parents in heterozygous state showed normal cardiac screening including cardiac evaluation, ECG, and echocardiography. A younger brother who was said to have similar systemic features was never referred to our center for scheduled cardiac screening.

## Discussion

Titinopathy is one of the most important causes of myopathy, which can be either congenital or of early or late-onset. Muscular and skeletal involvement is well described in the literature, and it can be associated with cardiac disorders especially in adults. There is extremely limited knowledge regarding the pediatric presentation of cardiac manifestations, and this is one of the cases in which the extracardiac aspect should be carefully considered during the work-up of children with cardiac disease leading to a comprehensive multisystemic diagnosis (17, 18).

The titin protein is organized into four structurally and functionally distinct regions that correlate with muscle sarcomere. These regions, located from the amino terminus to the carboxy terminus of the protein, include the Z-disk, I-band, A-band, and M-line (19). Different variants are responsible for the heterogeneous phenotype.

Missense *TTN* variants are frequent in the general population without a clear interpretation at the momentin terms of disease causality. A rapidly growing term is *TTNtv* which includes those variants with non-sense, splicing, and frameshift variants. TTNtv when correlated to specific interpretation of ACMG criteria, PSI, and clinical (both muscular and cardiac) features has a major clinical impact and is more described in late literature compared with the difficult interpretation of TTN missense variant (6).

The prevalence of heterozygous (monoallelic) *TTNtv* is estimated to be up to 2% in the general population (20, 21), whereas biallelic *TTNtv* are less frequently described, and their exact prevalence is not yet established. Several reports have shown that variants closer to the C-terminus lead to less severe cardiac involvement. However, different expression and severity of cardiac involvement may be related to other factors, including differences in the proportion of mutated titin isoform incorporated in the sarcomere, protein stability, or other modifying variants in *TTN* or myocardial-expressed genes (22).

Sporadic reports described biallelic N2BA/N2B variants with the highest risk of cardiac involvement in titinopathy, both in terms of age of onset and disease severity (20, 23). However, deep cardiac phenotyping, including clinical presentation, arrhythmic events, imaging, and histological myocardial findings, has rarely been detailed.

One of the very few literature data that described the multisystemic aspect of biallelic *TTNtv* was provided by Carmignac et al. (24) who reported on two consanguineous families (three and two patients in family 1 and 2, respectively). All patients died before the age of 20 years. One patient died suddenly at the age of 17.5 years despite normal cardiac screening, and the other four patients had early-onset DCM with severe LV systolic dysfunction and rhythm disturbances, including both tachyarrhythmias (polymorphic PVCs, ventricular tachycardia, atrio-ventricular reentrant tachycardia, premature atrial complexes) and bradyarrhythmias (first-degree AVB). No CMR data were reported likely because of the era in which the study was conducted (2007). The endpoint was reached in all patients: three patients died suddenly, and one patient died 2 years after HT. The study did not report details regarding medical therapy. In each family, homozygous titin deletion in exons encoding the C-terminal M-line region was confirmed. Functional studies confirmed that both deletions were caused by the presence of titin truncation (24). Other patients with associated congenital myopathy and early-onset DCM due to biallelic *TTNtv* have been reported by Savarese et al. and Oates et al., but only scant information about cardiac involvement was provided (6, 23). In our study, we report on five pediatric patients with congenital recessive titinopathy supported by genetic data, histopathological findings, and clinical features.

The referral to our center was due to cardiac reasons in two patients, cardiac and neuromuscular reasons at birth in one patient, and neuromuscular disorders with subsequent cardiac involvement in the remaining two patients. Cardiac symptoms were dyspnea and failure to thrive/loss of appetite in all cases. Male sex was more prevalent (four males vs. one female).

All patients showed cardiac involvement with severe LV dysfunction. Two patients required HT, whereas two other patients showed a good response to maximal conventional medical therapy and improved up to mild LV dysfunction.

HF conventional medical treatment can improve and decrease pulmonary wedge pressure, increase cardiac output, improve symptomatic status, and prevent disease progression.

In case of unrecovery, mechanic circulatory support and heart transplantation are the final options. Side effects of conventional medical treatment are not highly relevant in this setting, and in general all drugs are well tolerated. Hypotension and bradycardia are avoided by gradually titrating dosages and stopping it at the maximal tolerated dose (25).

Interestingly, all but one patient showed LGE on CMR. The LGE pattern was of non-ischemic type ranging from subepicardial to transmural LGE. This aspect is similar to that observed in other arrhythmogenic CMPs. CMR studies did not show pathognomonic LGE changes related to biallelic *TTNtv*. However, the arrhythmic aspect was not highly expressed: only one patient showed NSVT recorded on 24-h Holter ECG monitoring, two patients showed isolated PVCs with different morphologies and low arrhythmic burden, and in another patient, no arrhythmias were detected. Sinus rhythm was observed in all patients, nocturnal first-degree AVB occurred in two patients, and QTc at the upper limit of normal without pathological prolongation was recorded in two patients.

It is well known that *TTNtv* are associated with increased arrhythmic events, independent of conventional risk factors. However, our data show a low rate of arrhythmogenic events compared with other forms of DCM probably due to the young age of our patient cohort (26–31).

Other structural cardiac features include congenital heart defects. It is increasingly recognized that genetics play an important role in a wide spectrum of cardiac involvement including congenital heart defects, myocardial disorders, and arrhythmic disorders (32). Indeed, one patient (patient 1) had a small atrial septal defect, another patient (patient 5) underwent percutaneous closure of a large atrial septal defect, and the third patient (patient 2) had a surgically corrected large atrial and ventricular septal defect. Several reports in the literature described congenital heart defects in association with titinopathy in line with our findings (33) or in association with pulmonary valve stenosis, peripheral pulmonary stenosis, bicuspid aortic valve, and coarctation of the aorta (23).

Septal defects might represent a red flag in the setting of titinopathies in patients with congenital myopathies, though more data are needed to confirm this finding.

Muscular and skeletal characteristics showed common features. Generally, creatine kinase levels are normal in congenital myopathies characterized by titinopathy. Exceptionally, one of our patients showed constant hyperCKemia, and another patient had transient hyperCKemia after HT.

At the systemic level, other clinical features that may raise the suspicion of titinopathy include delay in motor development and ambulation, generalized hypotonia, generalized and diffuse pterygium-like lesions/semiflexed elbow and knee position, limited bending of the neck, foot and gait abnormalities, scoliosis or hyperlordosis, and rigid spine.

Dysmorphic features consist of long face, turricephaly, downslanting palpebral fissures, mild palpebral ptosis, low-set ears, plagiocephaly, prominent glabella and protruding eyes, eversion of lower palpebral fissures, synophria, flaring of eyebrows, high nasal bridge, long philtrum, cone-shaped teeth overcrowding, open bite, short neck, arachnodactyly, single palmar crease, bell-shaped thorax, narrow shoulders, and deformed toes.

Muscular MRI reveals systematically diffuse fatty infiltration of the legs and of the paravertebral and intercostal muscles. Muscle biopsy may be required during the diagnostic work-up and may be useful to confirm the diagnosis of primary myopathy.

The involvement of the respiratory muscles is also responsible for respiratory failure, abnormal cough reflex, and obstructive nocturnal hypo/apnea.

Reduced bone mineral density was also detected in three of our patients. Osteopenia and pathological bone fractures are reported in literature in this patient cohort, but the exact mechanism is still unknown. It might be thought that fixed-flexion joint movements, myopathy, and reduced mobility can play a major factor in this condition. However, further multicentric observations and metabolic studies are needed to undermine the specific nature of this feature (22).

## Conclusion

Biallelic *TTNtv* may cause severe and early-onset DCM. Compound heterozygous may result in a more severe and earlier-onset phenotype. This disorder can reach the attention of three main specialists in clinical practice: experts in neuromuscular diseases, pediatric cardiologists, and clinical geneticists. Red flags that can raise the suspicion of this specific genotype include both cardiac and extracardiac features. At the cardiac level, it can be associated with progressive pattern of cardiomyopathy, associated congenital septal defects (mainly septal), tachy- and bradyarrhythmias, and a non-ischemic LGE pattern on CMR. Arrhythmic risk stratification should be taken into account by performing regular 24-h Holter ECG monitoring, even though in pediatric age may be of limited value. End-stage heart failure is common in this patient population, and HT can be successfully performed if indicated. At a multisystemic level, skeletal and muscular abnormalities, such as arthrogryposis and congenital progressive myopathy, can be detected as early as in neonatal age. HyperCKemia is not a characteristic feature of titinopathy. For clinical geneticists, the observation of cardiomyopathy in a child with complex arthrogryposis and congenital progressive myopathy can represent a landmark and an indication to think about biallelic TTNtv.

A multidisciplinary evaluation of these patients should also include respiratory function tests, bone density evaluation, and family screening.

## Data Availability

The datasets presented in this study can be found in online repositories. The names of the repository/repositories and accession number(s) can be found in the article.
